# Biodegradation of polyvinyl chloride by *Citrobacter koseri* isolated from superworms (*Zophobas atratus* larvae)

**DOI:** 10.3389/fmicb.2023.1175249

**Published:** 2023-05-16

**Authors:** Indra Nyamjav, Yejin Jang, Ye Eun Lee, Sukkyoo Lee

**Affiliations:** ^1^Laboratory of Environmental Biotechnology, Department of Brain Sciences, Daegu Gyeongbuk Institute of Science and Technology (DGIST), Daegu, Republic of Korea; ^2^School of Undergraduate Studies, College of Transdisciplinary Studies, Daegu Gyeongbuk Institute of Science and Technology (DGIST), Daegu, Republic of Korea

**Keywords:** plastic biodegradation, polyvinyl chloride, *Citrobacter koseri*, gut microbiota, *Z. atratus*

## Abstract

Polyvinyl chloride (PVC) is one of the widely used plastic products worldwide, and its accumulation in the natural environment has become a major global issue with regard to the environment and biotic health. There is accordingly strong demand for the development of solutions and methods for environmental remediation. Degrading plastic waste using microorganisms is an effective and eco-friendly method. However, evidence of bacteria that afford efficient biodegradation of unplasticized, pure PVC film has yet to be reported. Therefore, the biodegradation of PVC becomes very important. Here, we present results on the physicochemical and structural studies of PVC by *Citrobacter koseri* (*C. koseri*) isolated from the gut of the superworm, *Zophobas atratus* (*Z. atratus*) larvae. We also studied the biodegradability of PVC by the gut microbiota compared with *C. koseri*. We analyzed the microbial degradation of the PVC surface using field emission scanning electron microscopy (FE-SEM) and energy-dispersive X-ray spectroscopy (EDS) and confirmed that the physical and chemical changes were caused by *C. koseri* and the gut microbiota. The chemical structural changes were further investigated using X-ray photoelectron spectroscopy (XPS) and Fourier-transform-infrared (FTIR) spectroscopy, and it was confirmed that the oxidation of the PVC surface proceeded with the formation of carbonyl groups (C = O), and hydroxyl groups (−OH) by *C. koseri*. Additionally, the gut microbiota composed of diverse microbial species showed equal oxidation of PVC compared to *C. koseri*. Further, we evaluated the capabilities of single bacterial isolate and gut microbiota for pure PVC film biodegradation. Our results verified that *C. koseri* and the culturable microbiota from the gut of superworms present similar potential to utilize pure PVC film as a carbon source. These findings provide a potential solution for the biodegradation of unplasticized PVC.

## Introduction

Environmental plastic waste pollution poses enormous ecological challenges. The global annual production of plastics was around 400 million metric tons for 2021, and this can be categorized into polyvinyl chloride (PVC) 10%, polypropylene (PP) 19.3%, low-density polyethylene (LDPE) 17.5%, high-density polyethylene (HDPE) 12.2%, polyurethane (PUR) 7.5%, polyethylene terephthalate (PET) 7.4%, and polystyrene (PS) 6.7%. PVC is the third most widely produced synthetic plastic material globally (Basmage and Hashmi, 2020). The total global production volume of PVC in 2018 amounted to 44.3 million metric tons (Kaushal et al., 2021).

Due to plastic’s limited recycling, long-term half-life, and physical compactness, millions of tons of plastic waste accumulate annually in the sea and spread out into the food chain (Danso et al., 2019). Only 40% of plastic waste is collected through collection systems; the other 60% ends up in natural environments (Borrelle et al., 2020). PVC is one of the most common polymer-based plastic pollutants reported worldwide (Danso et al., 2019; Ramkumar et al., 2022). Globally, waste plastic is commonly disposed of in landfills and incinerated (Lithner et al., 2011), which releases secondary pollutant compounds and extremely hazardous byproducts of Cl_2_, HCl, dioxins, and organochlorine compounds as PVC wastes are disposed of Lithner et al. (2011) and Hahladakis et al. (2018). Because of PVC’s broad range of industrial applications and low production prices, the pollution problem created by PVC products is considered a severe environmental issue (Danso et al., 2019). For this reason, alternative approaches must be developed for more sustainable and eco-friendly plastic waste management.

The discovery of PETase, a PET degrading enzyme, in soil bacteria (*Ideonella sakaiensis*) shed light on a biologically sustainable way of using plastic waste recycling technology (Yoshida et al., 2016). This discovery showed that plastic biodegrading strains and enzymes should be identified from nature. Other types of plastic biodegradation research are in the early stages of development. In this light, it is crucial to find more types of biodegrading bacteria, as such bacteria will have a growing number of applications.

Research into the biodegradation of PVC faces challenges due to PVC’s covalent backbone and the chlorinated vinyl groups in its molecular structure (Zhang et al., 2022). Moreover, additive- or plasticizer-free pure PVC is chemically resistant to alkalis, most acids, and organic solvents (Kotova et al., 2021). Several studies have reported solely fungal (*Aureobasidium pullulans, Polyporus versicolor, Phanerochaete chrysosporium ME446, Aspergillus niger*, and *Trichoderma hamatum*) and bacterial (*Pseudomonas citronellolis, Bacillus flexus*, and *Bacillus amyloliquefaciens)* PVC biodegradable species (Kirbaş et al., 1999; Webb et al., 2000; Ali et al., 2014; Giacomucci et al., 2019; Novotný et al., 2022). However, most of those studies used plasticized or pretreated PVC materials (Giacomucci et al., 2020; Peng et al., 2020) as a carbon source, which impeded a clear determination of the mechanism underlying pure PVC biodegradation abilities of such microbes. For this reason, any reduction in the weight of plastics exposed to microorganisms or modifications of their physical properties, such as flexibility or tensile strength, may be due to diminishing amounts of additives rather than to the degradation of PVC chains (Kotova et al., 2021). Unfortunately, comprehensive case studies of unplasticized PVC biodegradations have yet to be reported, and current knowledge of pure PVC-degrading microbes is limited.

In this study, we isolated gut microbiota from the superworms, larvae of *Z. atratus*, that was solely fed with PVC film. The superworms were surveilled, and the weight loss of PVC film was monitored throughout the consumption period. Further, we examined changes in their gut microbiota compositions using 16S rRNA amplicon sequencing, and among them, PVC-degradable bacteria were identified. Larvae gut microbial community analysis showed community shifts due to the PVC diet. We then compared isolated sole bacteria and whole gut microbiota biodegradation capacity on pure PVC film without pretreatment using FE-SEM, EDS, high-temperature gel permeation chromatography (HT-GPC), XPS, FTIR spectroscopy, proton nuclear magnetic resonance (^1^H NMR) spectroscopy, thermogravimetric analysis (TGA), and water contact angle measurement. The results demonstrate that *C. koseri* had a better or similar capacity than gut microbiota from superworms to degrade pure PVC film.

As a result of our findings, we demonstrated the possibility of biodegradation of PVC by isolated bacterial species, *C. koseri* and the gut microbiota *in vitro*. The research will enhance our understanding of PVC biodegradation and identify genetic mechanisms that regulate its pathway.

## Materials and methods

### PVC consumption by superworms and analysis of the gut microbial communities

The superworms were purchased from S-Worm (Cheonan, Korea) and were placed in a breeding chamber. The larvae were subject to a 48-h starvation period before initiating their experimental diets. We divided experimental diet conditions into PVC and bran consumption groups, respectively; each group consisted of 40 superworms in the 3–4 instar growth stage. It duplicated both experimental groups in independent breeding chambers, and totally we used 160 superworms larvae. For consumption, PVC films were sliced into 2 × 2 cm^2^ square sheets, and 2 g of PVC film (CV311200/3, unplasticized, 99.9% purity, Goodfellow, Huntingdon, UK), with 0.2 mm thickness, was supplied as the larvae’s sole carbon source in each of chamber. We bred them for 21 days in a cage with a temperature of 25°C and a humidity of 50–60%. At the end of the experiment, we selected six larvae from bran and PVC-exposed groups based on their movement ability and physical activity by visual perspective.

Intestinal samples were collected from the larvae using previously established technical procedures (Kim et al., 2020). In brief, the larvae were immersed in 70% ethanol and then washed with a sterile saline solution (0.9% NaCl) before their intestines were extracted. After the larvae’s heads and tails were removed, their intestines were extracted using a pair of sterilized forceps. The extracted guts were chopped and vortexed with 20 mL of saline solution for 5 min and then centrifuged for 8 min at 4,000 rpm to remove the epithelial cells. The operation was performed in a sterile environment. The total microbiota of the intestine extractions was enriched in 80 mL of nutrient broth (NB) medium (#234000, Difco Laboratories, Sparks, MD, USA) at 37°C for overnight culturing. These cultures were defined as the gut microbiota and aliquoted to DNA extraction, sequencing and further experiments.

Gut microbial communities of *Z. atratus* larvae were analyzed to investigate the difference in community structure between bran feeding and PVC feeding groups using the 16S rRNA gene amplicon sequencing. The microbial total genomic DNA was extracted and duplicated for each experimental group using a bacterial DNA isolation kit (Biosolution, Suwon, Korea) according to the manufacturer’s instructions. Following extraction, DNA was quantified (ng/μL) and assayed for quality (A260/A280 ratio is at ∼1.8 and A260/A230 ratio is at ∼2.0) using a NanoDrop UV visible spectrophotometer (NanoDrop 2000, Thermo Scientific, Boston, MA, USA) and DNA isolation quality was analyzed using 1% agarose gel electrophoresis at 100 V for 20 min. The DNA was stored in the dark at −20°C prior to sequencing. The V3-V4 region of the 16S rRNA gene of the sample was sequenced using Theragen Bio (Seongnam, Korea). A total of four samples were sequenced: two bran and two PVC. Library preparation of the V3–V4 hypervariable region of the 16S rRNA gene was performed according to the 16S Metagenomic Sequencing Library Preparation Illumina protocol (Part #15044223 Rev. B; Illumina, San Diego, CA, USA). The library pool containing equal molar quantities of each sample was sequenced using a MiSeq. 2 × 300 system (Illumina). The 16S V3-V4 sequencing reads were demultiplexed using the split_libraries_fastq.py function in the QIIME2 (2019.01 version) (Bolyen et al., 2019) metagenome analysis pipeline, and the sequences were quality trimmed using the Divisive amplicon denoising algorithm 2 (DADA2) pipeline in R (version 3.3.2). The set of unique V3-V4 sequences, referred to as amplicon sequence variants (ASVs), were then inferred using DADA2, and an ASV table of read counts per ASV per sample was generated. ASV was taxonomically classified using the sklearn-based Naïve Bayesian classifier (DeSantis et al., 2006) with the SILVA version 138 16S rRNA sequence database (Quast et al., 2013).

### Bacterial isolation and screening

To isolate a single species, the intestinal materials of six larval extracted guts were vortexed with 20 mL of sterile saline solution (0.9% NaCl) for 5 min and then centrifuged for 8 min at 4,000 rpm. The supernatant layer was then carefully transferred to a flask containing 80 mL of liquid carbon-free basal medium (LCFBM) (pH: 7.0) prepared with deionized water (per 1,000 mL) containing 0.7 g of KH_2_PO_4_, 0.7 g of K_2_HPO_4_, 0.7 g of MgSO_4_⋅7H_2_O, 1.0 g of NH_4_NO_3_, 0.005 g of NaCl, 0.002 g of FeSO_4_⋅7H_2_O, 0.002 g of ZnSO_4_⋅7H_2_O, and 0.001 g of MnSO_4_⋅H_2_O (Daejung chemicals & metals Co., Siheung, Korea) (Li et al., 2020) and 0.5 g of sterilized PVC film. The mixture solution was cultured for 2 months in a shaking incubator at a temperature of 25°C and a speed of 180 rpm under aerobic conditions.

At the end of 2 months of incubation, we used a micro-spray inoculation technique (Shin et al., 2021) with PVC-dissolved organic solvent and carbon-free basal agar medium. First, 0.5 g of PVC film was dissolved in 100 mL of tetrahydrofuran (THF) (Daejung chemicals and metals Co., Siheung, Korea) at 25°C. PVC solutions were then sprayed onto a carbon-free basal agar medium (1 L LCFBM containing 15 g agar) plate using a 0.35 mm nozzle with compressed air at a pressure of 150 kPa (Beetle bug, Korea). The organic solvent was evaporated in a fume hood for 1 h and sterilized under a UV-C lamp for 30 s. The PVC solution on the surface was completely dried on a clean bench overnight. The gut solution was diluted to 1/100 with LCFBM, and 2 mL of LCFBM solution containing bacteria was sprayed onto a plastic-coated agar plate using a micro-spray. The agar plate was incubated at 37°C until colony formation. Ten colonies were collected from each plate, and each colony was cultured in 3 mL of nutrient broth at 37°C overnight.

After bacterial growth, bacterial stocks were prepared from freshly grown cultures by mixing bacterial suspensions 1:1 with filter-sterilized glycerol in culture medium (50% vol/vol) and frozen at −80°C for long-term storage.

### Molecular identification of the bacteria

Further, genomic DNA was extracted using a genomic DNA extraction kit (Biosolution, Suwon, Korea) according to the manufacturer’s instructions. To identify the bacterial species, 16S rRNA gene was amplified using primers for 16S_27F (5′-AGAGTTTGATYMTGGCTCAG-3′) and 16S_1492R (5′-GGTTACCTTGTTACGACTT-3′). The reaction mixture was prepared by mixing AccuPower PCR premix [1X PCR buffer with 1.5 mM MgCl_2_, 250 μM dNTPs, 2.5 U Taq DNA polymerase (Bioneer, Daejeon, Korea)], 1 μL of 10 pmol of each primer, and 1 μL of DNA template (40 ng). The final reaction volume was completed to 50 μL using nuclease-free water (Biosolution, Suwon, Korea). PCR amplification was carried out in a T100 (Bio-rad, California, USA) thermal cycler, which was programmed for one cycle at 95°C for 10 min (initial denaturation), followed by 35 cycles of denaturation for 45 s at 95°C, annealing for 30 s at 46.5°C, extension for 90 s at 72°C, and a final extension for 5 min at 72°C. PCR products were displayed on 1 % agarose gel and photographed utilizing a gel documentation system. Molecular size of PCR products was determined in relation to 100 bp plus ladder (GenetBio, Daejeon, Korea) as a marker. PCR products were purified using a gel and PCR purification kit (Biofact, Daejeon, Korea), as described in the manufacturer’s manual. The purified PCR products of microbial gene fragments were sequenced at Macrogen sequencing company (Seoul, Korea) using an ABI 3100 automated sequencer with a Big Dye Terminator Kit version 3.1. The obtained sequences were compared with the NCBI database through BLAST searches.^1^ In this comparison, sequences of type species most closely related to the sequences of the isolates were searched. The gene sequences were assembled using BioEdit software version 7.2.5 (Hall, 1999).

### Bacterial growth rate on sole PVC

For the bacterial growth assay, we used M9 minimal medium consisting of M9 minimal salts (m-6030, Sigma-Aldrich, USA) supplemented with 1% glucose and 1% yeast extract. First, log phase *C. koseri* and gut microbiota were prepared by inoculating the culture at a 1:100 ratio in a NB medium and shaking at 200 rpm for 5–6 h at 37°C. The samples were then centrifuged at 4,000 rpm for 5 min and resuspended in M9 minimal medium. The remaining medium was entirely removed by repeating the same process two times. The starting point was the cell concentration obtained at OD600 = 0.1 and 1.0 g of PVC sheets (2 × 2 mm^2^) was then added to 30 mL of M9 minimal medium in a 90 mm glass petri dish at 37°C and cultured in a static incubator for 30 days. Non-inoculated M9 minimal medium as a positive control, which include the same amount of PVC sheets, was used in the growth rate experiments. The growth of the cells was measured at optical density at 600 nm (OD600) every 5 days for a duration of 30 days.

### PVC weight loss measurement

Isolated *C. koseri* and gut microbiota were cultured in a NB medium for 5–6 h, and the bacteria were resuspended in LCFBM after centrifugation. The remaining medium was entirely removed by repeating the same process two times. The recovered cells (1 × 10^8^ CFU) were added with 1.0 g of PVC sheets (2 × 2 mm^2^) to 30 mL of LCFBM containing 1% NB medium in a 90 mm glass petri dish at 37°C and cultured in a static incubator for 30 days. After treatment, the remaining PVC sheets were collected using a 70 μm cell strainer to quantify the weight loss of the PVC sheets. The PVC sheets were washed with 2% sodium dodecyl sulfate (SDS) (Junsei chemicals, Tokyo, Japan) for 4 h, rinsed with distilled water three to four times, and then dried in an air oven at 60°C for 24 h. The gravimetric weight loss was recorded independently in triplicate and expressed as a percentage.


$$\mathrm{Percentage}\,\mathrm{degradation}=\frac{\mathrm{Initial}\,\mathrm{weight}-\mathrm{Final}\,\mathrm{weight}}{\mathrm{Initial}\,\mathrm{weight}}\times 100$$

### PVC film incubation with *C. koseri* and microbiota for characterization analysis

*Citrobacter koseri* and the gut microbiota (1 × 10^9^ CFU) were sprayed onto both sides of a 2.5 × 2.5 cm^2^ PVC film using a micro-sprayer. The PVC films were incubated on LCFBM agar in a 6-well plate at 37°C for 30 days. The PVC films incubated on LCFBM agar for 30 days without bacteria were used as a control group. The PVC films were washed with 2% SDS for 4 h, rinsed with distilled water three to four times, and then dried in an air oven at 60°C for 24 h. The PVC films were used for further analysis, including FE-SEM, EDS, FTIR spectroscopy, ^1^H NMR, XPS, TGA, contact angle meter measurement, and HT-GPC.

The surface morphology of the PVC film was subsequently examined via FE-SEM (SU8230, Hitachi, Tokyo, Japan). After cutting the PVC film into a size of 1 × 1 cm^2^, *C. koseri* and microbiota were incubated with the PVC film on LCFBM agar for 30 days, respectively. After incubation, 2 % SDS solution was used for 4 h to remove bacteria attached to the film’s surface and then rinsed with distilled water three to four times. The film was fixed on copper tape, and a platinum coating was deposited via ion sputtering for 15 s at 15 mA (E-1045, Hitachi High-Technologies Corporation, Tokyo, Japan). The degraded plastic surface was then imaged at 3.0 kV acceleration voltage.

Energy dispersive X-ray spectroscopy with a module attached to the SEM (X-max 150, Oxford Instruments, Oxford, UK) was used to determine the elemental composition change of the PVC plastic film surface during *C. koseri* and gut microbiota mediated plastic biodegradation. Areas exhibiting proliferated *C. koseri* and gut microbiota were observed to show elemental composition change. Plastics without bacteria were used as control groups to analyze differences compared to the test groups. A composition analysis was performed via energy-dispersive X-ray spectroscopy with an acceleration voltage of 3.0 kV.

The surface chemical modification of the PVC films treated with *C. koseri* and the gut microbiota was analyzed using FT-IR. The potassium bromide (KBr) pellet method was used as follows. The PVC film was dissolved in THF and maintained in a glass vial at 25°C for 5 min to obtain a final concentration of approximately 2 mg/mL. The solvent was then evaporated using nitrogen gas for 6 h. Afterward, the samples were milled and compacted at room temperature for 1 min at a pressure of 6 MPa using a 13 mm diameter pellet die and a digital hydraulic presser (CrushIR, Pike Technologies, WI, USA). The pellets were prepared by mixing 1–2 mg of the samples with 100 mg of KBr. The chemical changes of the plastic film pellets were characterized by FT-IR (Nicolet 6700, Thermo Fisher Scientific, Waltham, MA, USA) in transmission mode with a scan range between 4000 and 500 cm^–1^ and a scan resolution of 4 cm^–1^ by collecting 16 scans for a single spectrum at ambient temperature. The obtained spectra were evaluated using OMNIC software, version 7.0 (Thermo Fisher Scientific, Waltham, MA, USA).

^1^H NMR experiments were performed on Bruker Avance III 400 spectrometer (Bruker, Germany) equipped with a 5 mm observe broadband probe head (BBFO) with z-gradients. The PVC film (5 mg) was dissolved in tetrahydrofuran-*d8* (99.5 atom %, Sigma-Aldrich, USA) solution (0.5 mL) and then transferred to NMR tubes for analysis. The spectra were reported in parts per million (ppm) and then recorded ^1^H NMR spectra were analyzed using TopSpin software, version 4.1.4 (Bruker, Germany).

Changes in elemental composition on the plastic surfaces were examined by measuring the binding energy with an X-ray photoelectron spectroscopy (ESCALAB 250Xi, Thermo Fisher Scientific, Waltham, MA, USA). For sample preparation, pre-arranged PVC film (1 × 1 cm^2^) fixed on the carbon tape was measured within an energy range of 276–300 eV, C1s, and 188–215 eV, Cl2p.

TGA was performed to detect the thermal decomposition of the samples using a thermogravimetric analyzer (Q500, TA Instruments, New Castle, DE, USA). Approximately 5 mg PVC films were tested from 20 to 800°C with a heating rate of 10°C/min. The tests were carried out in a nitrogen atmosphere (purity > 99.9%) with a flow rate of 60 mL/min. The obtained data were analyzed using Universal Analysis 2000 software, version 4.5A (TA Instruments, New Castle, DE, USA).

The molecular weight of PVC films was determined using the HT-GPC (EcoSEC HLC-8420 GPC, Tosoh Bioscience, Tokyo, Japan) with a differential RI detector and Shodex HK-G + 2× TSKgel Supermultipore HZ-M + TSKgel SuperHZ-2500 column, employing THF as the eluent with a flow rate of 0.35 mL per minute at 40°C. Twelve polystyrene standards with molecular weights ranging from 162 to 2,327,000 g/mol^–1^ were used for calibration. The sample concentration was 3 mg/mL, of which 20 μL was injected into the HT-GPC analyzer.

During PVC degradation by *C. koseri* and microbiota, a water contact angle analysis was performed to analyze changes in hydrophobicity on the PVC surface. First, the PVC film (1 × 1 cm^2^) was fixed on a carbon tape to measure the contact angle using a water contact angle meter (Phoenix Multi, Seoul, Korea), and 35 μL of water was dropped onto the PVC surface within a 20 mm^2^ area at 20°C. Both left and right contact angles were measured, and the mean value was used for the comparative analysis. All measurements were done in triplicate.

## Results

### *Z. atratus* larvae consumption on PVC film and gut microbial diversity

**FIGURE 1:**
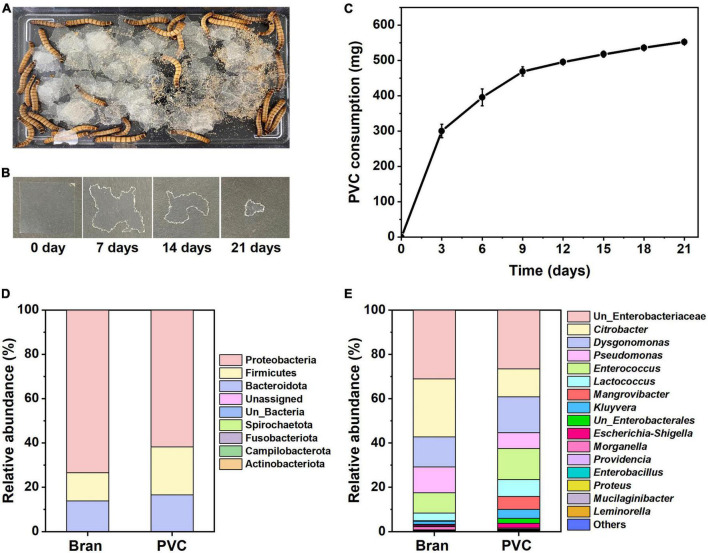
*Z. atratus* larvae consumption on PVC film and comparison of gut microbial communities. PVC film feeding behavior of *Z. atratus* larvae and time intervals of PVC film ingestion by superworm **(A,B)**. Superworm consumption rate (*n* = 40) on PVC film **(C)**. The PVC film weight was measured every 3 days for 21 days to determine the rate of reduction by superworms. Forty superworms consumed about 0.552 ± 0.079 mg of PVC film over 21 days. Gut microbiota composition of the *Z. atratus* larvae at the phylum **(D)** and genus **(E)** level.

We characterized the ingestion rate of the superworms after feeding with PVC as a sole diet (Figures 1A, B). Forty superworms consumed approximately 552 mg of PVC film over 21 days (Figure 1C).

The PVC consumption rate of the larvae was 26.3 mg per day, and the amount of plastic ingested by the superworms is associated with the weights of the worms, and consumption rates per superworm are also presented in Table 1. The average consumption rate was 13.8 mg per superworm for 21 days. These findings indicate that the sole PVC film could provide an energy source and sustain the survival of the *Z. atratus* larvae.

**TABLE 1:** PVC consumption rate by superworms.

Consumption rate per day (mg) by 40 superworms	Consumption rate per worm over 21 days (mg)
26.3 ± 3.76	13.8

The gut microbiota should be essential for PVC film degradation by *Z. atratus* larva. We defined the bacteria isolated from the gut, and they survived in the NB medium as cultural gut microbiota because we cultured the gut extract in the NB medium for 1 week. The culturable gut microbiota of the *Z. atratus* larvae was analyzed to study the differences in community structure using high-throughput 16S rRNA gene-based sequencing between bran-fed and PVC-fed groups (Figures 1D, E). The purpose of this experiment was to compare the microbial community profiles before and after PVC feeding to superworms. Previous studies suggest that the gut microbiota shifted to different patterns of microbial community after plastics ingestion (Przemieniecki et al., 2020; Luo et al., 2021; Zhang et al., 2022).

16S rRNA gene amplicon sequencing analysis of the superworm gut microbiota showed three major abundant phyla showed variations between the bran-fed and PVC-fed group, including Proteobacteria, Firmicutes and Bacteroidota (Figure 1D) at the phylum level. In this study, the relative abundance of predominated Proteobacteria decreased (73.5–61.7%), while the co-dominated Firmicutes (12.6–21.7%) and Bacteroidota (13.7–16.4%) increased in larvae fed with PVC film. Of 40 identified genera, 16 were relatively abundant, including unclassified *Enterobacteriaceae, Citrobacter, Dysgonomonas*, etc. (Figure 1E). Among major genera, there was a high abundance of unclassified *Enterobacteriaceae, Citrobacter, Dysgonomonas, Pseudomonas*, and *Enterococcus* in the bran-fed group. In contrast, PVC-fed larvae microbiota consisted of various new groups of the bacterial genus, such as unclassified *Enterobacterales, Mangrovibacter, and Leminorella*. This suggests that PVC film biodegradation shifted the larvae’s gut microbiota composition, which caused microbial cross-communication of metabolic interactions. In summary, 16S rRNA amplicon sequencing results demonstrated that the culturable gut microbiota between the bran and PVC diets in superworms differed in terms of community structure.

### Identification of PVC-degrading bacterial species

After culturing the gut extract from the superworms for 2 months without any treatment, ten colonies were screened, and their bacterial 16S rRNA was sequenced. All bacterial colonies were identified as *C*. *koseri* (NCBI accession ID: OQ678054) by 16S rRNA sequencing analysis (Supplementary Figure 1). In the *in vivo* condition, complex interactions of different microbial species of gut microbiota may drive PVC biodegradation and the 2 months period of PVC enrichment culture acted as a stress to select only the most survival species *in vitro*, such as *C*. *koseri*.

### Growth of *C. koseri* and gut microbiota on PVC film and PVC mass changes

**FIGURE 2:**
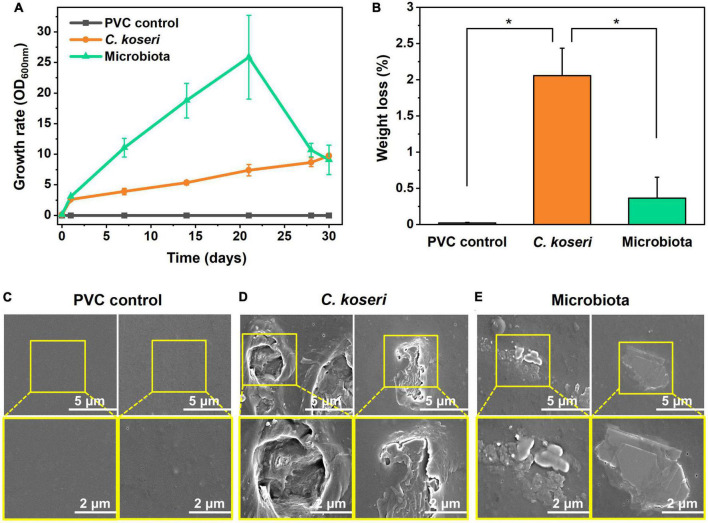
Biodegradation effects of PVC films by *C. koseri* and gut microbiota and film surface modification. Comparison of growth rates of *C. koseri* and microbiota supplied with PVC film as the sole carbon source **(A)**. Weight loss of PVC film mediated by *C. koseri* and microbiota isolated from the gut of superworms **(B)**. *C. koseri* and microbiota showed PVC biodegradation of 2.06 and 0.36%, respectively. Significant differences among the three groups were determined by a Tukey’s pairwise test (*p ≤ 0.05). Comparison of FESEM micrographs of PVC control **(C)** and treatment with *C. koseri* **(D)** and microbiota **(E)**.

Plastic biodegradation is associated with the growth rate of bacteria in carbon-free medium with plastics as the sole carbon source. The growth rate of *C. koseri* in M9 minimal medium with sole PVC sheets gradually increased. Culturable microbiota populations presented more efficient growth kinetics in M9 minimal medium until 20 days. The growth declined in the latter days of the culture period, which might be due to bacterial secondary metabolite accumulation in the batch culture. The gradual increase of optical density of the cells demonstrates the biodegradation activity of *C. koseri* and gut microbiota, suggesting that they utilized PVC sheets in their metabolism as a carbon source. In contrast, the positive control was not changed through the cultural periods (Figure 2A).

Furthermore, we assessed the weight loss of the PVC sheets by gravimetry as the most straightforward technique, with bacterial cells thoroughly removed from the sheet, and the weight percentage showed decreased results after 30 days of treatment (Figure 2B). The weight loss of the PVC sheets was estimated at 2.06 and 0.36%, incubated with *C. koseri* and whole gut microbiota, respectively. In contrast, the control group of the PVC sheet without a bacterial treatment sample was determined to have undergone 0.02% weight loss. The weight reduction and bacterial growth kinetics results suggest that both treatment groups, *C. koseri* and microbiota, can use PVC as a carbon energy source in carbon-free culture conditions.

### Changes in the surface morphology and properties of PVC film after *C. koseri* and microbiota treatment

We next assessed whether physical changes in the surface properties of the PVC film caused the weight reduction of the film, which was characterized by FE-SEM (Figures 2C–E). Scanning electron microscopy results show apparent biodegradation of the PVC film after *C. koseri* and microbiota treatment, including roughness, erosion, and cracks appearing on the film surface (Figures 2C, D). In contrast, the control group’s results showed no visible biodegradation of the PVC film under the same conditions (Figure 2E).

**FIGURE 3:**
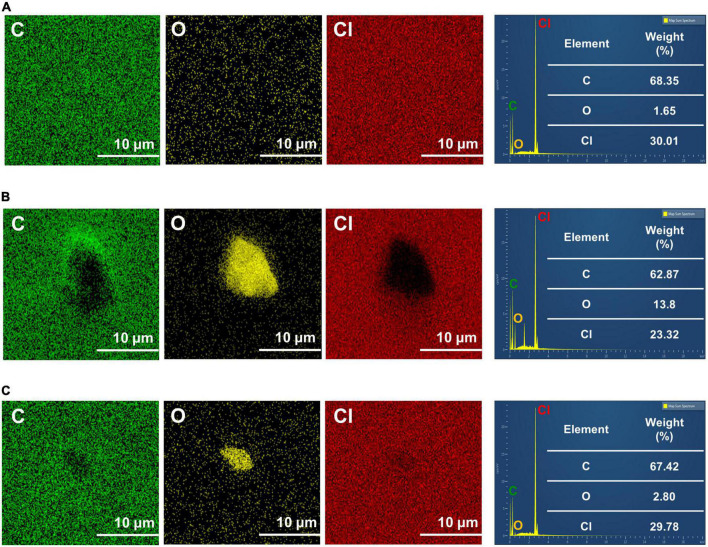
Energy dispersive X-ray spectroscopy mapping of C, O, and Cl of PVC film. The results are for untreated PVC control **(A)**, *C. koseri* treatment **(B)**, and gut microbiota treatment **(C)** after 30 days.

Elemental analysis results of the PVC surface using EDS are shown in Figures 3A–C. The weight percentage of carbon content in the PVC control was 68.35%, whereas that of the PVC incubated with *C. koseri* was 62.87%, and the microbiota showed a value of 67.4%, respectively. The weight percentage of oxygen content in the PVC control was 1.65%, whereas the PVC incubated with *C. koseri* and the gut microbiota showed values of 13.8 and 2.80%, respectively (Figure 3). The chloride content was 30.01% in the PVC control and 23.32% in PVC cultured with *C. koseri*. In summary, oxidation content was significantly increased on the PVC film after treatment with *C. koseri* and the gut microbiota. In contrast, carbon and chloride content was decreased compared with the control group, suggesting that oxidation proceeds more actively on the surface of the PVC film incubated with *C. koseri* and microbiota groups.

### Verification of PVC degradation effects by chemical characterization analysis

**FIGURE 4:**
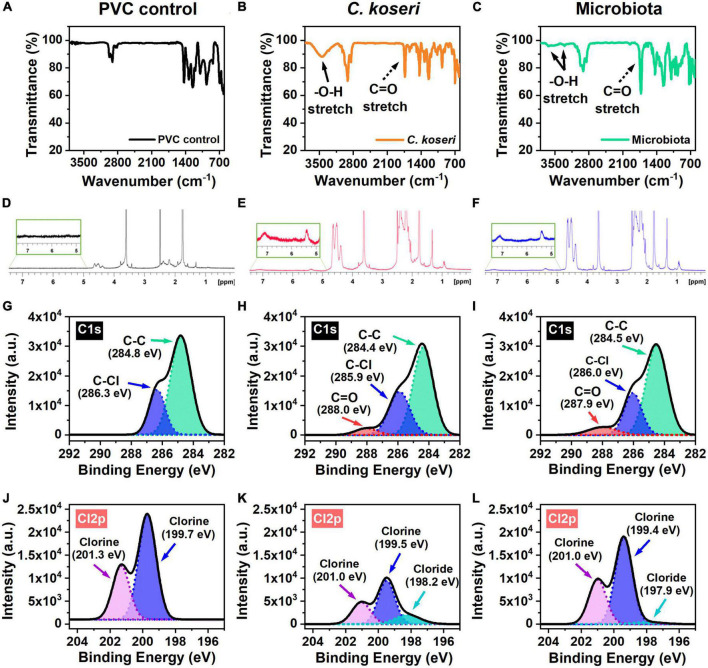
Chemical characterization of the PVC film. FT-IR spectra of PVC films biodegraded by *C. koseri* indicated by the orange line, gut microbiota the green line, and versus the control the black line **(A–C)**. Solid black arrows denote the hydroxyl group (O–H); dashed black arrows the carbonyl group (C = O). The ^1^H-NMR results of PVC film in *C. koseri*, gut microbiota, and untreated control **(D–F)**. XPS spectra of the C1s **(G–I)**, and Cl2p **(J–L)** of the PVC film samples: PVC control (left column), *C. koseri* treatment (middle column), and microbiota treatment (right column).

Chemical changes of PVC by the *C. koseri* during biodegradation were validated using FTIR spectroscopy. The FTIR spectra of the control group and the bacterial treatment by microbiota and *C. koseri* are presented in Figures 4A–C. Figure 4A shows the FTIR spectra of the PVC film control sample, which does not appear to have oxidative peaks. In contrast, the spectra of the PVC film inoculated with *C. koseri* revealed new functional group peaks at 1,735 cm^–1^ corresponding to a carbonyl group (C = O) and another new broadband between 3,100 and 3,600 cm^–1^ associated with a hydroxyl group (O-H), respectively (Figure 4B). The spectra of the PVC film incubated with the gut microbiota present a small new functional hydroxyl group (O-H) peak between 3,350–3,500 cm^–1^ and new functional group peaks at 1,731 cm^–1^ corresponding to a carbonyl group (C = O) (Figure 4C). The appearance of carbonyl and hydroxyl groups is regarded as a preliminary and indispensable step during the oxidation and depolymerization of plastics (Peng et al., 2019, 2020). These findings suggest that both bacterial treatment groups with *C. koseri* and microbiota have the potential to influence PVC oxidation in carbon-free culture media.

^1^H NMR experiments were carried out to obtain information on the structures of the PVC film after treatment with *C. koseri* and microbiota culture in LCFBM (Figures 4D–F). The ^1^H NMR spectra of PVC in *C. koseri* and microbiota showed that some substances had been newly produced in the position of 5.0∼7.5 ppm, suggesting the products have a functional group of alkene (−C = C-), whereas this was not observed in the control group. These results suggest the new formation of a functional alkene group after treatment due to depolymerization of the PVC film, which is consistent with several prior studies (Brandon et al., 2018; Peng et al., 2020; Zhang et al., 2022).

An XPS analysis was performed to independently verify FTIR data to investigate changes in the chemical compositions of the PVC films. XPS spectra of C1s showed that the surface elemental composition of the PVC films (Figures 4G–I). The C1s spectrum of the PVC control only showed double peaks at 284.8 eV and 286.3 eV, which are attributed to the C-C and C-Cl bond in the long chain of the PVC structure (Figure 4G). The spectra of the PVC films incubated with *C. koseri* and microbiota showed new additional peaks at 288.0 eV and 287.9 eV, corresponding to carbonyl groups (C = O) (Figures 4H, I). The presence of oxygen in PVC is due to the oxidation of the polymer chain (Beamson and Briggs, 1992). The Cl2p spectrum of the PVC control showed chlorine peaks at 201.3 eV and 199.7 eV, whereas the spectra of the PVC films incubated with *C. koseri* and microbiota, revealed new chloride peaks at 198.2 eV and 197.9 eV, respectively (Figures 4J–L). Cl2p peaks decrease after *C. koseri* and microbiota treatment due to the dechlorination of the PVC film. The relative abundance of the C = O peak of PVC films incubated with *C. koseri* and the gut microbiota is summarized in Table 2. These results suggest that the chemical composition of the PVC film changed after incubation with *C. koseri* and the gut microbiota, respectively. The XPS results are consistent with the FTIR and EDS results.

**TABLE 2:** Peak areas (%) of C1s, and Cl2p of surface layer of PVC film were estimated using XPS analysis.

Sample	C1s peak area (%)			Cl2p peak area (%)		
	**C-C/** **C-H**	**C-Cl/** **C-O**	**O-C = O**	**Cl 2p_3/2_** **chloride**	**Cl 2p_1/2_** **chlorine**	**Cl 2p_3/2_** **chlorine**
PVC control	73.39	26.61	0	0	67.17	32.83
*C. koseri*	62.47	32.49	5.04	17.2	53.43	29.37
Gut microbiota	66.58	25.91	7.51	2.53	63.89	33.58

### Reduction of thermal stability, hydrophobicity, and molecular weight of PVC film

**FIGURE 5:**
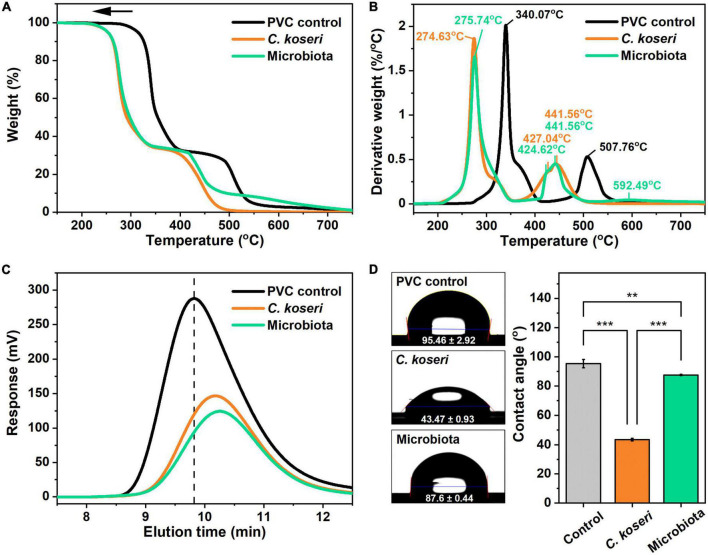
Thermal stability, hydrophobicity, and molecular weight analysis of PVC film by *C. koseri* and gut microbiota after 30 days. Thermogravimetric analysis curves of weight loss **(A)** and corresponding derivative weight **(B)** of the PVC film. Comparison of gel permeation chromatography elution profiles of control versus treatment groups **(C)**. Water droplets (left column) and values of water contact angles (right column) on PVC film: untreated control, *C. koseri* treatment, and microbiota treatment **(D)**. The statistical significance of the contact angle plot was determined by a one-way ANOVA followed by a paired comparison test (**p ≤ 0.01; ***p ≤ 0.001).

Thermal modifications of PVC films after *C. koseri* and whole microbiota treatments were determined using TGA in a nitrogen environment. Figures 5A, B show the mass loss and the corresponding derivative of mass loss curves of the PVC control and PVC films treated with *C. koseri* and gut microbiota at a heating rate of 10°C/min. Two maximum decomposition rates appeared in the PVC control group at 340.07°C and 507.76°C. For the *C. koseri* treatment group, three different maximum decomposition rates were detected at 274.63, 427.04°C, and 441.56°C, while microbiota treated groups, four different maximum decomposition rates were detected at 275.74°C, 424.62°C, 441.56°C, and 592.49°C, respectively. The maximum decomposition rates shifted compared with the PVC control group, indicating that the thermal stability of the PVC film significantly declined due to the PVC polymer chain shortening or breaking during treatment with *C. koseri* and microbiota.

We subsequently ran an HT-GPC analysis to examine the molecular weight of the PVC film after 30 days of treatment with *C. koseri* and microbiota and a control group without bacterial inoculation. The molecular weight distributions of the PVC films are shown in Figure 5C, which illustrates that the elution band of the PVC control group was at 9.82 min. In contrast, elution bands of films treated with *C. koseri* and microbiota were at 10.26 and 10.18 min, respectively. The elution band value was shifted toward a higher value after the bacterial treatment, which resulted in larger PVC molecules eluting from the column sooner and decreasing the retention time. In contrast, the smaller PVC molecules elute later, resulting in a longer elution time during the HT-GPC analysis. These results indicate that PVC films were fragmented, and their molecular weight decreased during both bacterial treatments. The decreases in the molecular weight of the PVC treated with both groups indicated that *C. koseri* and gut microbiota could depolymerize PVC into smaller fragments. Moreover, it can be attributed to the action of exocellular enzymes released into the culture medium, which causes hydrolysis of polymers at the ends of backbone chains and within the chains (Wilkes and Aristilde, 2017).

Next, we measured the wettability of PVC, a naturally hydrophobic material, according to the PVC water contact angle after different bacterial treatments with *C. koseri* and whole microbiota. The water contact angle on untreated PVC was 95.46°, whereas with the treatment of *C. koseri* it was 43.47°, and the microbiota treatment group presented a value of 87.6° (Figure 5D). On the other hand, treatment of *C. koseri* and microbiota decreased the value of the surface water contact angle by 51.99° and 7.86°, respectively. This result indicates that the PVC’s hydrophobic surface property was changed to be more hydrophilic, which could allow contact with more water molecules in the solution environment. In addition, increased hydrophilicity promotes the attachment of microorganisms to the polymer surface, further accelerating the PVC degradation rate (Gabisa and Gheewala, 2022).

## Discussion

As noted in the introduction, a limited number of PVC-degradable microbes have been reported in the literature. In this work, we have isolated PVC-degrading bacteria from the gut of superworms that were fed only unplasticized PVC films as a food source using conditional starvations. We found that the superworms can digest PVC films and maintain the survival of the larvae stage caused by an energy source derived from PVC-degradable bacteria in the intestinal microbiota. We then isolated *C. koseri* species that can degrade PVC in cultural conditions. In addition, we tested the bacterial synergetic effect of PVC biodegradation using whole microbiota extract from the superworm gut.

Next, we evaluated their biodegradation abilities using conventional microbial cultural techniques combined with spectroscopic studies such as FE-SEM, FTIR, ^1^H NMR, TGA, XPS, EDS, HT-GPC analysis and surface contact angle meter measurement on the PVC film. Substantial surface damage was observed in the scanning electron microscopic investigations of the *C. koseri* and microbiota inoculated PVC films. The surface of the inoculation-free PVC film maintained its original smooth and clear surface morphology. Furthermore, using spectroscopic approaches we verified that such surface damage is associated with chemical oxidation of the main chain of PVC.

Based on the chemical signature and building blocks of PVC, the PVC biodegradation process entails multistage chemical conversions: (a) depolymerization or breaking down polymer chains, (b) formation of oxidized intermediates, and (c) mineralization of intermediates to CO2, H2O, and Cl-, when PVC is the degradation target (Peng et al., 2020). In the present study, oxidized functional groups were identified through multiple independent spectroscopic tools; for instance, the vibrational fingerprint of oxidized carbonyl carbon (C = O) and hydroxyl (C-OH) groups were determined by FTIR and the electronic binding energy of oxidized carbon of Cs1 was described by XPS. Also, spectroscopic results were confirmed using an elemental analyzer coupled with scanning electron microscopy. Together these results verified that newly generated functional groups consist of oxidized parts in the PVC film, and these results demonstrate the initiation of biodegradation in our samples due to bacterial growth. Interestingly, sole bacterial inoculation and microbiota treatment both showed chemical modifications in unplasticized PVC films.

As we mentioned above, the first stage of biodegradation is the depolymerization of PVC. To evaluate this, we examined depolymerization of PVC film using HT-GPC and TGA, which are widely used as an indication to characterize depolymerization and thermal stability. The HT-GPC analysis provided changes in molecular weight, and the TGA analysis showed that the thermal stability of the PVC film was decreased, which verified the depolymerization and decomposition of the PVC film during the bacterial cultural period. On the other hand, the films lose thermal stability and become fragile due to bacterial attack during the PVC bio-fragmentation process (Yuan et al., 2020). Moreover, the plastic film’s structural decomposition and oxidation are associated with its surface wettability and hydrophobicity. Due to this, we used a water contact angle meter to test the surface wettability of PVC films that were treated with *C. koseri and* microbiota as well as the untreated control film. Consequently, the hydrophobic surface of the PVC film was rendered hydrophilic by *C. koseri* and microbiota treatment, which increases cell attachment on the film surface. Contact angle measurement results supported the morphological and spectroscopic analyses of PVC films.

Overall, understanding PVC films’ structural and chemical changes in bacterial treatments shall further enhance PVC biodegradation studies. It has also been speculated that the oxidation stage plays an important role in PVC biodegradation, and the discovery of oxidizer enzymes might be a key point of further studies.

## Conclusion

This study verified that *C. koseri* isolated bacteria from the gut of superworms was capable of PVC biodegradation in carbon-free culture conditions. Interestingly, the *C. koseri* was presented equal or better capability with culturable gut microbiota for PVC biodegradation *in vitro*. Our findings suggest that both experimental groups were able to modify the morphology and structure of the unplasticized PVC film. Further studies are required to elucidate the exact mechanism of PVC biodegradation and its catalytic target enzymes.

## Data availability statement

The datasets presented in this study can be found in online repositories. The names of the repository/repositories and accession number(s) can be found below: NCBI–OQ678054.

## Author contributions

IN performed visualization, investigation, data curation, and wrote the first draft of the manuscript. YJ and YL performed visualization and investigation. SL performed conceptualization, methodology, supervision, and reviewed the manuscript. All authors contributed to the article and approved the submitted version.

## Footnotes

1
https://blast.ncbi.nlm.nih.gov/Blast.cgi
