# Supplementary material for: Biodegradation of polyvinyl chloride by Citrobacter koseri isolated from superworms (Zophobas atratus larvae)
Source: Front Microbiol. 2023 May 16;14:1175249. doi: 10.3389/fmicb.2023.1175249 (PMC10228827; doi:10.3389/fmicb.2023.1175249)
Supplement: Supplementary file 1 [file Image_1.pdf]

Supplementary Material

Biodegradation of polyvinyl chloride by *Citrobacter koseri* isolated from superworms (*Zophobas atratus* larvae)

Indra Nyamjav[1], Yejin Jang[2], Ye Eun Lee[1,] Sukkyoo Lee[1]*

*** Correspondence:** Sukkyoo Lee
slee2012@dgist.ac.kr

GCGCATGGGCGGGCAAGCCTACACATGCAGTCGAACGGTAACAGGAAGCAGCTTGCTG
CTTCGCTGACGAGTGGCGGACGGGTGAGTAATGTCTGGGAAACTGCCTGATGGAGGGGG
ATAACTACTGGAAACGGTAGCTAATACCGCATAACGTCGCAAGACCAAAGAGGGGGAC
CTTCGGGCCTCTTGCCATCAGATGTGCCCAGATGGGATTAGCTTGTTGGTGGGGTAACGG
CTCATCCAAGGCGGACGATCCCTAGCTGGTCTGAGAGGATGACCAGCCACACTGGAACT
GAGACACGGTCCAGACTCCTACGGGAGGCAGCAGTGGGGAATATTGCACAATGGGCGC
AAGCCTGATGCAGCCATGCCGCGTGTATGAAGAAGGCCTTCGGGTTGTAAAGTACTTTC
AGCGGGGAGGAAGGTGTTGTGGTTAATAACCGCAGCAATTGACGTTACCCGCAGAAGA
AGCACCGGCTAACTCCGTGCCAGCAGCCGCGGTAATACGGAGGGTGCAAGCGTTAATCG
GAATTACTGGGCGTAAAGCGCACGCAGGCGGTCTGTTAAGTCAGATGTGAAATCCCCGG
GCTCAACCTGGGAACTGCATCTGATACTGGCAGGCTTGAGTCTCGTAGAGGGGGGTAGA
ATTCCAGGTGTAGCGGTGAAATGCGTAGAGATCTGGAGGAATACCGGTGGCGAAGGCG
GCCCCCTGGACGAAGACTGACGCTCAGGTGCGAAAGCGTGGGGAGCAAACAGGATTAG
ATACCCTGGTAGTCCACGCCGTAAACGATGTCGACTTGGAGGTTGTGCCCTTGAGGCGT
GGCTTCCGGAGCTAACGCGTTAAGTCGACCGCCTGGGGAGTACGGCCGCAAGGTTAAAA
CTCAAATGAATTGACGGGGGCCCGCACAAGCGGTGGAGCATGTGGTTTAATTCGATGCA
ACGCGAAGAACCTTACCTGGTCTTGACATCCACGGAAGTTTTCAGAGATGAGAATGTGC
CTTCGGGAACCGTGAGACAGGTGCTGCATGGCTGTCGTCAGCTCGTGTTGTGAAATGTT
GGGTTAAGTCCCGCAACGAGCGCAACCCTTATCCTTTGTTGCCAGCGGTTAGGCCGGGA
ACTCAAAGGAGACTGCCAGTGATAAACTGGAGGAAAGGTGGGGATGACGTCAAGTCAT
CATGGCCCTTACGACCAGGGCTACACACGTGCTACAATGGCATATACAAAGAAGAAGCG
ACCTCGCGAGAGCAAGCGGACCTCATAAAGTATGTCGTAGTCCGGATTGGAGTCTGCAA
CTCGACTCCATGAAGTCGGAATCGCTAGTAATCGTGGATCAGAATGCCACGGTGAATAC
GTTCCCGGGCCTTGTACACACCGCCCGTCACACCATGGGAGTGGGTTGCAAAAGAAGTA
GGTAGCTTAACCTTCGGGAGGGCGCTACCACTTTGGATTCTTATGTCTT

Supplementary Figure 1. The 16s rRNA gene sequence generated for *C. koseri* had 1459 bases.
